# Spatial Perspective-Taking in Children With Autism Spectrum Disorders: The Predictive Role of Visuospatial and Motor Abilities

**DOI:** 10.3389/fnhum.2020.00208

**Published:** 2020-06-03

**Authors:** Ramona Cardillo, Cristiana Erbì, Irene C. Mammarella

**Affiliations:** Department of Developmental and Social Psychology, University of Padua, Padua, Italy

**Keywords:** spatial perspective-taking, neurodevelopmental disorders, autism spectrum disorder, visuospatial abilities, motor abilities

## Abstract

Despite its impact on everyday functioning, spatial perspective-taking has rarely been investigated in autism spectrum disorders (ASD), and previous findings are surprisingly sparse and inconsistent. In the present study, we aimed to investigate spatial perspective-taking abilities in children and adolescents with ASD without intellectual disabilities, comparing them with a group of typically developing (TD) peers. Our objectives were: (i) to test similarities and differences between these groups in a spatial perspective-taking task; and (ii) to see whether similar or different underlying processes (i.e., fine and gross motor skills, and visuospatial abilities) might account for the groups’ performance in the spatial perspective-taking task. A group of children with ASD (*N* = 36) was compared with a TD group (*N* = 39), aged from 8 to 16 years. Participants were administered tasks assessing spatial perspective-taking, fine and gross motor skills, visuo-constructive abilities, visuospatial working memory, visual imagery, and mental rotation. Our results revealed that the ASD group had more difficulty with the spatial perspective-taking task than the TD group. The two groups also had some shared and some different processes that predicted their perspective-taking performance: a significant predictive effect of fine motor skills and visuospatial working memory emerged for both groups, while gross motor skills (i.e., walking heel-to-toe) and visuospatial imagery only revealed a role in the TD group. These findings suggest that different abilities might account for the two groups’ performance in the spatial perspective-taking task. Gross motor skills and complex visuospatial abilities seem to be more important in sustaining spatial perspective-taking ability in typical development than in the event of ASD. Some of the clinical and educational implications of these findings are discussed.

## Introduction

Autism spectrum disorders (ASD) are characterized by deficits in social communication, social interaction, and obsessive/stereotyped patterns of behavior, interests or activities (American Psychiatric Association, 2013). Other, non-social factors also have an important role in the cognitive profiles of children with ASD (Cardillo, 2018), even for those with no intellectual disabilities (ID). One of the features of the cognitive phenotype of this disorder is an atypical perceptual processing, particularly for complex visual stimuli (Caron et al., 2006; Cardillo et al., 2018). A vast amount of research on the role of these processing peculiarities in the visuospatial domain in individuals with ASD has revealed a heterogeneous profile of strengths and weaknesses, depending on the type and complexity of the tasks administered (e.g., Edgin and Pennington, 2005; Happé and Frith, 2006; Kuschner et al., 2007; Mammarella et al., 2019; Cardillo et al., 2020). The crucial role of visuospatial functioning in ASD emerges clearly from its possible consequences on everyday life and adaptive behaviors. Visuospatial abilities are essential to interaction with the environment (Hegarty and Waller, 2005; Jansen and Heil, 2010) and involved in many daily activities, from navigating in the environment to recognizing and manipulating objects, to recalling locations (Tzuriel and Egozi, 2010; Cardillo, 2018). From the academic standpoint, visuospatial skills predict success in science, technology, engineering, and math (Humphreys et al., 1993; Uttal and Cohen, 2012; Andersen, 2014; Khine, 2017; Mammarella et al., 2018). Visuospatial abilities can be trained (Uttal et al., 2013a, b; Meneghetti et al., 2017), so it is fundamentally important to understand the factors that influence performance on measures of these skills (Schmidt et al., 2013; Tarampi et al., 2016).

One of the crucial components of the multi-faceted construct of visuospatial ability is spatial perspective-taking (Eilam and Alon, 2019), which involves a higher-level, conscious, and deliberate mental transformation that corresponds to the spatial orientation factor (Thurstone, 1950; Huttenlocher and Presson, 1973; Lohman, 1988; Kessler and Rutherford, 2010). Spatial perspective-taking consists in seeing a space from a different perspective, adopting new imaginary orientations, mentally viewing a scene from an external viewpoint (Pearson et al., 2013). This spatial transformation process occupies a crucial place at the convergence of perception and mental imagery (Kessler and Rutherford, 2010). It is particularly important in “large-scale” spatial activities, when individuals can imagine “being part of” or “move through” a space (Münzer et al., 2018). In fact, tasks investigating spatial perspective-taking abilities have revealed an important role in predicting people’s environment-learning (Allen et al., 1996; Pazzaglia and De Beni, 2006), navigating and wayfinding abilities (Kozhevnikov et al., 2006).

One task that enables spatial perspective-taking abilities to be investigated is the Object Perspective-Taking Test (OPT) developed by Kozhevnikov and Hegarty (2001) and Hegarty and Waller (2004). This test assesses an individual’s ability to mentally adopt new imaginary positions within a configuration of objects. It was developed to better explore the distinction between spatial orientation and spatial visualization performance, or the ability to make egocentric and object-based spatial transformations, respectively (Meneghetti et al., 2012). Hegarty and Waller (2004) confirmed that a distinction could be drawn between these two spatial factors using a confirmatory factor analysis in which the perspective-taking factor was dissociated from mental rotation. Despite this dissociation, these two factors proved to be strictly related (Kozhevnikov and Hegarty, 2001; Hegarty and Waller, 2004). Specifically, Hegarty and Waller (2004) using different measures of perspective-taking and mental rotation abilities, showed that these two spatial factors were highly correlated (*r* = 0.80), indicating that they have a consistent portion of shared variance. In order to account for this shared variance, authors suggested different hypotheses. First, perspective taking and mental rotation may rely on common processes (i.e., encoding and memory of spatial images). Second, participants might use the same strategy to perform both perspective taking and mental rotation tasks. Third, similar innate or environmental factors might influence one’s ability to solve the two types of spatial transformations (Hegarty and Waller, 2004).

Given the complex nature of spatial perspective-taking, some published studies investigated the role of different factors underlying people’s performance. Meneghetti et al. (2012) showed that OPT performance is sustained by specific spatial abilities and by the use of different strategies. The authors administered the OPT and several visuospatial tasks and self-report measures to undergraduate students to investigate whether different spatial abilities and strategies sustained their OPT performance. The results showed that OPT performance was positively associated with spatial visualization ability and a preference for spatial imagery strategies, while it was negatively associated with the use of a mental rotation strategy.

Visuospatial working memory and motor abilities have also been found to have an important influence on spatial perspective-taking performance of children and adults (Kaiser et al., 2008; Eilam and Alon, 2019). Neuroimaging studies, conducted with adults, showed activation of areas involved in general cognitive control processes (such as working memory) and the supplementary motor area during the execution of a mental rotation task or a spatial perspective-taking task (Johnston et al., 2004; Kaiser et al., 2008). In particular, Kaiser et al. (2008), found the activation of the supplementary motor area in healthy adults during the execution of a spatial perspective-taking task. Authors highlighted that the activation of this brain region can relate to the encoding of the stimuli in relation to the observer, as well as to the cognitive processes involved in the perspective transformation. Only few studies have explored the relationship between perspective taking and motor abilities in children. Newcombe and Frick (2010) suggested that the developmental progress of perspective taking abilities is strictly related to motor development, and motor activity has been found to facilitate children’s performance in this kind of tasks. According to the authors, it would seem that children’s mental spatial transformation abilities can profit from active movements, by allowing them to draw into consolidated links between action and cognition (Newcombe and Frick, 2010). In addition, children’s perspective taking skills were found to be related with their spatial drawing abilities, which involve visuo-motor skills (Ebersbach et al., 2011). However, to the best of our knowledge, no research has explored the relationship between spatial perspective taking and motor abilities in children with neurodevelopmental disorders.

In addition, despite its impact on everyday functioning and strong association with various visuospatial abilities, spatial perspective-taking in ASD has been investigated only rarely (David et al., 2010), and with inconsistent results (Pearson et al., 2013). Some studies involving adults or/and children, found spatial perspective-taking performance intact in participants with ASD, and concluded that any deficits in this area were not crucial in the ASD profile (Hobson, 1984; Reed and Peterson, 1990; Tan and Harris, 1991; David et al., 2010). Others reported evidence of poor spatial perspective-taking abilities in children with this clinical diagnosis (Yirmiya et al., 1994; Warreyn et al., 2005). Some authors argued that a possible explanation for the discrepant findings across studies lies in the different tasks administered (i.e., items vs. appearance questions) and the way the instructions were presented (i.e., viewer vs. object-rotation instructions) (Langdon and Coltheart, 2001; David et al., 2010). Considering the tasks, item questions ask to judge which object in an array of features occupies a specific position relative to another viewpoint, while appearance questions ask how an array would appear from another perspective (Langdon and Coltheart, 2001). Concerning the instructions, in the viewer rotation the examinee is asked to imagine moving himself relative to a fixed array, while in the object rotation is asked to imagine rotating an array relative to the viewer fixed position (Langdon and Coltheart, 2001). According to David et al. (2010), adults with ASD seem to perform better on item questions, particularly when they have to manage with viewer rotation instructions (i.e., “Which object would be to your right if you were in that position?”) while, employing object rotation instructions (i.e., “Which object would be to your right if we turned the stand so that side over there were in front of you?”) would be disadvantageous for this clinical group. Thus, the use of different tasks and instructions could explain discrepant findings across studies.

As for motor skills, to our knowledge no research has investigated their role in predicting the spatial perspective-taking performance of participants with ASD. However, previous studies involving adults or/and children extensively reported poor fine and gross motor skills in individuals with ASD (Ming et al., 2007; Fournier et al., 2010; Staples and Reid, 2010; Whyatt and Craig, 2012). Differences in these underlying processes should therefore be taken into account when considering the variability in spatial perspective-taking performance of individuals with ASD.

The findings described thus far highlight the need to analyze the spatial perspective-taking abilities of individuals with ASD in more depth. Only a handful of studies have explored these spatial skills in such individuals, and none investigated the concurrent role of both visuospatial and fine and gross motor skills. The present study aimed to investigate spatial perspective-taking abilities in children and adolescents with ASD without ID comparing them with a group of typically developing (TD) peers. Participants were administered tests to measure their fine and gross motor skills, visuo-constructive abilities, visuospatial working memory, visual imagery and mental rotation.

To clarify the similarities and differences between the two groups’ spatial perspective-taking performance, our first aim was to seek possible differences in terms of angular disparity. To establish whether similar or different underlying processes might account for the groups’ performance in the spatial perspective-taking task, we also used two separate models: one for the role of fine and gross motor skills in predicting spatial perspective-taking performance; the other for the involvement of visuospatial abilities (i.e., visuo-constructive abilities, visuospatial working memory, visuospatial processing, and mental rotation).

Although we expect some differences between groups, the inconsistency of previous reports on the spatial perspective-taking abilities of individuals with ASD (Pearson et al., 2013) prevented us from making any specific predictions regarding our groups’ performance. Given the role of motor abilities and visuospatial factors underlying spatial perspective-taking, we might expect to find a significant effect of visuospatial imagery (Meneghetti et al., 2012), visuospatial working memory, and motor abilities (Johnston et al., 2004; Kaiser et al., 2008; Meneghetti et al., 2016) in sustaining our participants’ perspective-taking performance.

## Materials and Methods

### Participants

The study involved 75 participants aged between 8 and 16 years old: 36 (34 M) children with ASD but no ID, and 39 (36 M) matched TD controls. The two groups did not statistically differ in chronological age [*F*(1, 73) = 0.34, *p* = 0.563; *R*^2^*_adj_* = 0.009], gender distribution [χ^2^(*df* = 1) = 0.008, *p* = 0.926], or total IQ [*F*(1, 73) = 1.34, *p* = 0.250; *R*^2^*_adj_* = 0.018]. A summary of the participants’ characteristics is shown in Table 1.

**TABLE 1:** Characteristics of the two groups: children with autism spectrum disorders but no intellectual disability (ASD); and typically developing (TD) peers.

Measures	ASD (*n* = 36)	[Min–Max]	TD (*n* = 39)	[Min–Max]	*F*(1, 73)	*P*	Cohen’s *d*
Gender (M:F)	34:2		36:3				
**Age (year; month)**							
Mean (*SD*)	10;10 (2;8)	[8;0–16;10]	11;3 (2;10)	[8;00–16;8]	0.337	0.563	0.13
**IQ^*a*^**							
Mean (*SD*)	98.30 (12.75)	[80–135]	101.62 (11.97)	[83–132]	1.34	0.250	0.27
**ADI-R: A**							
Mean (*SD*)	15.77 (7.26)	[10–29]	4.05 (3.64)	[0–9]	80.03	<0.001	2.04
**ADI-R: B**							
Mean (*SD*)	11.64 (5.07)	[8–23]	2.85 (2.12)	[0–7]	98.61	<0.001	2.27
**ADI-R: C**							
Mean (*SD*)	6.61 (3.05)	[3–14]	1.41 (0.75)	[0–2]	87.59	<0.001	2.36

**^*a*^Standard scores on the Wechsler Intelligence Scale for Children – Fourth Edition. IQ, Intelligence Quotient; ADI-R, Autism Diagnostic Interview (Rutter et al., 2005); ADI-R: A, Reciprocal Social Interaction; ADI-R: B, Language/Communication; ADI-R: C, Repetitive Behaviors/Interests. Higher scores on the ADI-R reflect more severe autistic symptoms.**

All participants were recruited via local community contacts, at specialized centers (for children with ASD), or schools (for the TD children).

Children in the ASD group had all received an independent clinical diagnosis according to the DSM-IV-TR (American Psychiatric Association, 2000) or ICD-10 (World Health Organization, 1992) criteria. They also scored above the cut-off for ASD in the Autism Diagnostic Interview – Revised (ADI-R; Rutter et al., 2005). Children with ASD were only included in this study if they achieved a standard score of 85 or more for total IQ on the Wechsler Intelligence Scales (WISC IV: Wechsler, 2003).

The TD group consisted of healthy children of normal intelligence with no history of psychiatric, neurodevelopmental or neurological disorders. In addition, having a family member with a neurodevelopmental disorder was an exclusion criterion for this group. They were tested individually at school.

All participants spoke Italian as their native language and had no neurological, visual or hearing impairments. The study was approved by the research ethics committee at the University of Padua, Italy, and all parents had given prior written consent to their children’s participation by signing an informed consent form.

### Materials

#### Spatial Perspective-Taking

**FIGURE 1:**
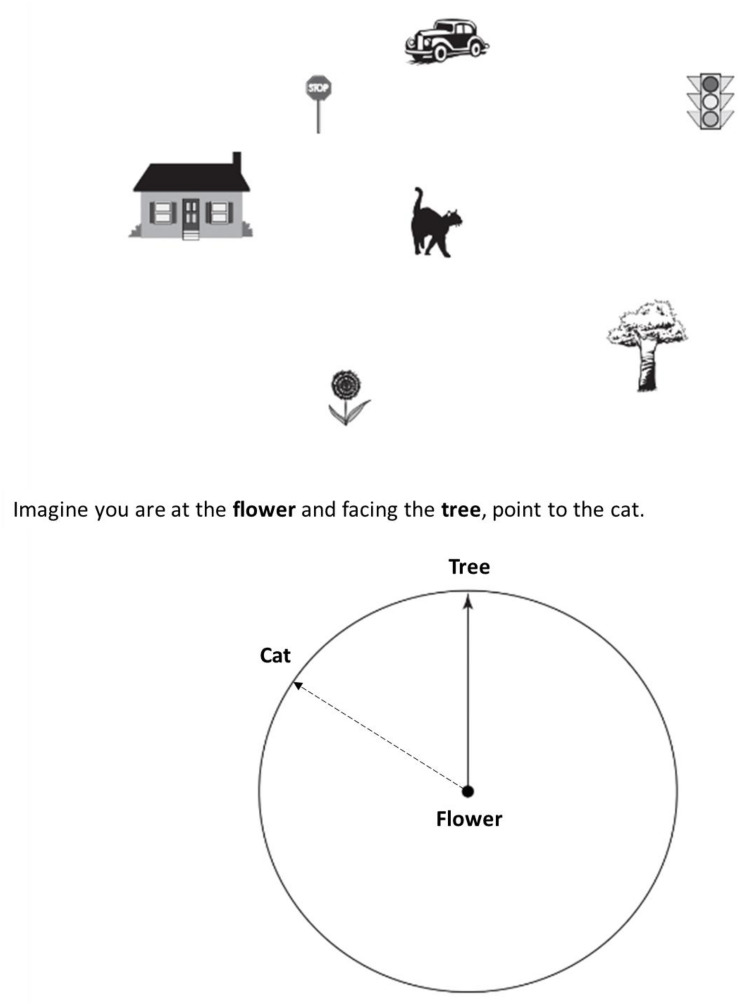
Example of an item in the Short Object Perspective-Taking task (sOPT). The dashed arrow indicates the correct response to the item (direction to the cat).

The *Short Object Perspective-Taking* (sOPT) task (adapted from Kozhevnikov and Hegarty, 2001; Hegarty and Waller, 2004) is a paper-and-pencil task comprising six items, each containing a configuration of seven objects drawn on the top half of an A4 piece of paper and a circle for the answer placed at the bottom half of the same page (see Figure 1). On each item, participants were asked to imagine being at one object in the layout (the station point), facing another (imagined heading), and pointing to a third (target object). Participants were asked to give their answers using the circle provided at the bottom half of the page, which displayed the station point (e.g., the flower) in the center of the figure, and the imagined heading (e.g., the tree) drawn as an arrow pointing vertically up. Participants were asked to draw an arrow from the center toward the edge of the circle, indicating the direction to the target object (e.g., the cat). An item example is reported in Figure 1; the dashed arrow indicates the correct response to the item. The time limit for completing the task was 5 min. The six items were divided into three categories, depending on the angular disparity with respect to the respondent’s point of view (0–60°, 60–120°, 120–180° in the right or left half-disk). The answers of two of the items fell in each of the three categories. The score corresponded to the deviation in degrees between the participant’s response and the correct direction to the target, for each item (degrees of error or angular disparity). The higher the degrees of error the worse the performance.

#### Fine and Gross Motor Abilities

Fine and gross motor abilities were analyzed using four subtests of the Movement ABC-2 (Henderson et al., 2007), two each from the Manual dexterity and Balance domains, respectively. Manual dexterity refers to the fine motor control of hands and fingers needed to manipulate objects. Dynamic balance involves gross motor skills that are specific goal-directed movement patterns. The following tasks were administered, and according to the manual, the version for the younger children (7–10 years old), and for the older ones (11–16 years old) was used:

#### Manual Dexterity 1 (MD 1)

Participants were asked to insert (younger children) or rotate (older children) 12 pegs in a pegboard. Children were asked to take the pegs one at time and to put them in the pegboard as soon as possible. The task was performed first with the dominant hand and then with the non-dominant hand. The task was timed and two trials were given for each hand; the best trial for each hand was used to rate the task. Response times were considered for scoring purposes.

#### Manual Dexterity 3 (MD 3)

Participants had to draw a trail between the two lines of a path of variable size (wider for the younger children, narrower for the older ones). Only the dominant hand was considered. A maximum of two trials were given, and the best trial was used to rate the task. If the child completed the first trial without errors, the second trial was not required. The number of errors was considered.

#### Dynamic Balance 1 (BAL 1)

Participants were asked to walk forward heel-to-toe (the younger children) or backward toe-to-heel (the older ones) on a 4.5 m long strip of adhesive tape placed on the floor. A maximum of two trials were given, children had to walk up to 15 steps or to the end of the line, and the best trial was used to rate the task. If the child completed the first trial without errors, the second trial was not required. The number of correctly completed steps was recorded.

#### Dynamic Balance 2 (BAL 2)

Participants were asked to hop on one-foot straight forward (the younger children) or zig-zagging from side to side (the older ones). Participants were required to jump five consecutive jumps on mats first with the dominant leg and then with the non-dominant leg. A maximum of two trials were given for each leg. If the child completed the first trial without errors, the second trial was not required. The best trial for each leg was used to rate the task and the number of hops completed was recorded.

For each task of the Movement ABC-2, raw scores (accuracy, errors or response times, depending on the task) were compared with normative values, and Scaled scores (*M* = 10, *SD* = 3) were computed. A scaled score from 1 to 7 is described as a below average score, a scaled score from 8 to 12 is described as an average score, finally a scaled score from 13 to 19 is described as an above average score.

#### Visuo-Constructive Abilities

The Rey-Osterrieth Complex Figure Test (ROCFT; Rey, 1941, 1968) is a neuropsychological test measuring visuo-constructive skills. Participants were asked to copy from the original figure a complex geometrical figure. To perform the copy condition, the stimulus figure was placed in front of the examinee, with the request to copy the figure as accurately as possible. The standard scoring system (Rey, 1968) was used to measure the accuracy of their drawing, awarding different scores (from 0 to 2) to each of the 18 elements comprising the figure depending on their presence or absence, and/or correct location in a participant’s drawing. There were not time limits for drawing the figure. The raw scores were considered for each participant. The higher the score the better the performance.

#### Visuospatial Working Memory

Two computerized tasks, adapted from Mammarella et al. (2018), were used to measure simultaneous and sequential spatial working memory. Each task consisted of a maximum of 21 items administered with a self-terminating procedure. Participants were shown a 5 × 5 grid and asked to memorize a number of cells presented simultaneously or sequentially. After 3 s, the initial stimulus was removed, and participants were shown a blank grid in which they had to reproduce the previously seen pattern of cells. In the spatial-simultaneous matrices (SSM), participants were asked to recall the position of the stimuli, while in the spatial-sequential matrices (SSQM), they needed to recall the stimuli in their order of presentation. In both tasks, the number of cells presented in each grid ranged from 2 to 8. The accuracy was calculated as a proportion i.e., the number of correct responses out of the total number of items performed. The higher the score the better the performance.

#### Visuospatial Processing

The Arrows task is a subtest of the Nepsy-II battery (Korkman et al., 2007), which assesses the ability to create and manipulate a mental representation of an object, and the ability to judge line orientation. The task consisted of 21 items. For each item participants looked at an array of arrows placed around a target and indicate the arrows that were pointing to the center of the target. The number of correct responses were considered and one point was awarded for each correct arrow detected. The scores obtained by each participant were compared with the normative values and expressed as scaled scores.

#### Mental Rotation

The Animal Rotation task derived from Kaltner and Jansen (2014) is a paper-and-pencil task used to assess mental rotation abilities. Participants were asked to look at a target figure and choose the corresponding figure from among four rotated options presented alongside. The stimuli consisted of 2D figures of animals, and the task included 21 items. Participants had 5 min to complete the task. One point was awarded for each correct response. The accuracy was calculated as a proportion, i.e., the number of correct responses out of the total number of items. The higher the score the better the performance.

### Procedure

Participants were tested in a quiet room during two individual sessions lasting ~40 min each. The tasks were administered in a counterbalanced order. Instructions were given for each task, and participants practiced with each task before starting the experiment. The computerized tasks were administered using a laptop computer with a 15-inch LCD screen.

### Data Analyses

Data were analyzed using R (R Core Team, 2015). First, the scores obtained from the sOPT task were modeled using a mixed-effects approach, and run using the “lme4” package (Bates et al., 2015). Both fixed and random effects were considered by means of a series of likelihood ratio tests for nested models based on the chi-square distribution (Pinheiro and Bates, 2000). For each model, the Akaike Information Criterion (AIC; Akaike, 1974) was reported and a lower AIC indicated a better model. The analyses were conducted considering every single trial for each participant and participants were included as random effects to consider their variability in the mixed-effect model. The following fixed effects and their interactions were tested: Group (2 levels: ASD, TD) and Angular disparity (3 levels: level 1 = 0–60°, level 2 = 60–120°, level 3 = 120–180°).

Then two different linear regression analyses were run to investigate the association between the dependent variable (sOPT) and the motor or visuospatial abilities considered, and to identify the most predictive combinations. First the measures of fine and gross motor abilities were included as predictors (i.e., Manual dexterity 1, MD 1; Manual dexterity 3, MD 3; Dynamic balance 1, BAL 1; Dynamic balance 2, BAL 2). Then the tasks measuring visuospatial abilities were considered (i.e., ROCFT; SSM; SSQM; Arrows; Animal Rotation). For both models, the main and interactive effect of Group (i.e., ASD, TD) was included as well (see Table 2 for the descriptive statistics of each measure by group).

**TABLE 2:** Means (*M*) and standard deviations (*SD*) by group: children with autism spectrum disorders but no intellectual disability (ASD); and typically developing (TD) peers.

Tasks		ASD (*n* = 36)		TD (*n* = 39)		*Cohen’s d*
		*M* (*SD*)	[Min–Max]	*M (SD)*	[Min–Max]	
sOPT degrees of error	Level 1	83.9 (52.83)	[5–180]	66.33 (53.55)	[0–171]	0.33
	Level 2	94.04 (55.26)	[1–176]	91.33 (57.79)	[0–177]	0.05
	Level 3	110.08 (65.44)	[0–180]	92.65 (67.52)	[2–180]	0.26
MD 1		4.72 (3.19)	[1.00–12.00]	5.95 (3.58)	[1.00–13.00]	0.36
MD 3		4.77 (4.11)	[1.00–12.00]	7.77 (4.03)	[1.00–13.00]	0.73
BAL 1		8.25 (3.93)	[1.00–12.00]	10.87 (1.87)	[4.00–12.00]	0.85
BAL 2		8.66 (3.96)	[1.00–12.00]	10.28 (2.42)	[4.00–12.00]	0.49
ROCFT		18.94 (8.53)	[4.50–32.00]	25.37 (6.26)	[11.50–35.00]	0.86
SSM		0.19 (0.22)	[0.01–0.99]	0.22 (0.16)	[0.03–0.70]	0.16
SSQM		0.15 (0.15)	[0.01–0.81]	0.18 (0.13)	[0.03–0.48]	0.21
Arrows		26.47 (8.24)	[4.00–38.00]	29.21 (3.78)	[16.00–38.00]	0.43
AR		0.75 (0.29)	[0.10–1.00]	0.76 (0.26)	[0.24–1.00]	0.04

**sOPT, Short Object Perspective-Taking task; MD1, Manual dexterity 1; MD3, Manual dexterity 3; BAL1, Dynamic balance 1; BAL2, Dynamic balance 2; ROCFT, Rey-Osterrieth Complex Figure Test; SSM, Spatial-Simultaneous Matrices; SSQM, Spatial-Sequential Matrices; AR, Animal Rotation.**

Additional analyses (differences between groups for each motor and visuospatial measure, correlations and skewness and kurtosis for the residuals of each regression model) were reported in section “Supplementary Material.”

We adopted a model selection strategy for all the variables examined (as in Fox, 2008, for example), following the same procedure to detect the best-fitting model. First, starting from the full model (M0 – which included the main effects of motor or visuospatial tasks, and their interaction with the effect of Group), we built the various models by subtracting one effect at a time, so that all the possible models were fitted. Then the models were compared using the Akaike Information Criterion (AIC, Akaike, 1974) as a fit index following the procedure suggested by Burnham et al. (2011), where the best model coincided with the smallest AIC. The best model(s) were selected from the set of models tested by applying information-theoretic (I-T) approaches, considering the AIC and the relative likelihood (*l*) of each model (Burnham et al., 2011). The values of AIC_s_, Δ^0^ AIC_s_ [Δ^0^ AIC = AIC*_f__ull_* – AIC*_i_*], ΔAIC*_s_* [ΔAIC = AIC*_bes__t__model_* – AIC*_i_*], and *l*_s_ [*l* = exp(ΔAIC/2)] were computed for each model: Δ^0^ AIC greater than 0 meant that a particular model *i* fitted the data better than the full model; ΔAIC described the distance between the best model and the other models computed; *l* values greater than 1 indicated that the model considered was more plausible. Details of the selected models and the indexes guiding model selection are given in Table 3.

**TABLE 3:** Model comparison investigating the association between the sOPT (dependent variable) and motor or visuospatial tasks (predictors).

Models		AIC	Δ°*AIC*	ΔAIC	*l*	Adjusted *R*^2^
**Motor skills**						
M0	sOPT ∼ Group (MD1 + MD3 + BAL1 + BAL2)	826.65	0	-4.38	0.11	0.18
M1	sOPT ∼ MD1 + Group*MD3 + Group*BAL1 + Group*BAL2	824.68	1.97	-2.41	0.30	0.19
M2	sOPT ∼ MD1 + BAL2 + Group*MD3 + Group*BAL1	823.08	3.57	-0.81	0.67	0.20
**M3**	sOPT ∼ MD1 + Group*MD3 + Group*BAL1	**822.27**	**4.38**	**0**	**1**	**0.20**
**Visuospatial abilities**						
M0	sOPT∼ Group (Arrow + ROCFT + AR + SSM + SSQM)	806.88	0	-6.57	0.04	0.38
M1	sOPT∼ SSM + Group*Arrow + Group*ROCFT + Group*AR + Group*SSQM	805.08	1.8	-4.77	0.09	0.39
M2	sOPT∼ SSM + SSQM + Group*Arrow + Group*ROCFT + Group*AR	803.76	3.12	-3.45	0.18	0.40
M3	sOPT∼ ROCFT + SSM + SSQM + Group*Arrow + Group*AR	803.24	3.64	-2.93	0.23	0.39
M4	sOPT∼ ROCFT + AR + SSM + SSQM + Group*Arrow	802.36	4.52	-2.05	0.36	0.39
M5	sOPT∼ ROCFT + SSM + SSQM + Group*Arrow	800.81	6.07	-0.5	0.78	0.40
**M6**	sOPT∼ SSM + SSQM + Group*Arrow	**800.31**	**6.57**	**0**	**1**	**0.40**

**AIC, Akaike Information Criterion; Δ°AIC, difference in AIC with respect to the full model (M0); ΔAIC, difference in AIC; l, relative likelihood with respect to best target model [i.e., exp(ΔAIC/2)]. The higher the ΔAIC and the adjusted R^2^, the better the model. sOPT, Short Object Perspective-Taking task; MD1, Manual dexterity 1; MD3, Manual dexterity 3; BAL1, Dynamic balance 1; BAL2, Dynamic balance 2; ROCFT, Rey-Osterrieth Complex Figure Test; AR, Animal Rotation; SSM, Spatial-Simultaneous Matrices; SSQM, Spatial-Sequential Matrices.**

Graphical effects were obtained using the “effects” package (Fox, 2003).

#### Group Differences in the Short Object Perspective-Taking (sOPT) Task

**FIGURE 2:**
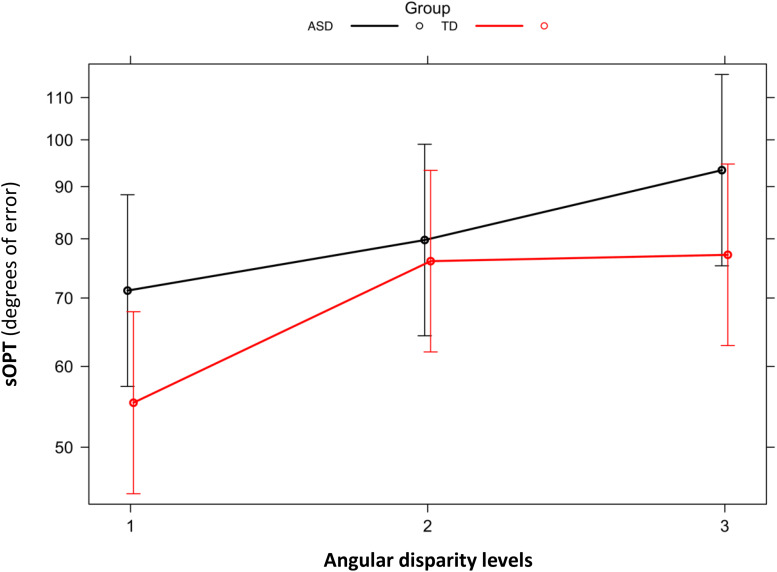
Short Object Perspective Taking task (sOPT). Degrees of error by group (ASD, TD) and level of angular disparity. Error bars represent 95% confidence intervals. ASD, autism spectrum disorder; TD, typically developing; sOPT, Short Object Perspective-Taking task; level 1 = 0–60°; level 2 = 60–120°; level 3 = 120–180°.

No main effect of Group emerged for the sOPT task [χ^2^(1) = 1.09, *p* = 0.30 (full model: *AIC* = 13,849; model without Group: AIC = 13,848)], but the main effect of the angular disparity was significant [χ^2^(2) = 614.23, *p* < 0.001 (model without Angular disparity: AIC = 14,459)]. The model coefficients showed that participants’ performance was more accurate for level 1 than for levels 2 and 3 (*p*s < 0.001), and it was more accurate for level 2 than for level 3 (*p* < 0.001). The analysis also revealed a significant interaction between Group and Angular disparity [χ^2^(2) = 71.469, *p* < 0.001 (model with interaction: AIC = 13,781)] (see Figure 2). The model coefficients showed that the ASD group’s performance was less accurate than the TD group’s on level 1 (*p* = 0.04), while the groups did not differ on levels 2 and 3 (*p* = 0.77 and 0.11, respectively). The group with ASD showed significant differences between the various levels of angular disparity: their performance was more accurate for level 1 than for levels 2 and 3, and it was more accurate for level 2 than for level 3 (*p*s < 0.001). The group with TD also showed significant differences in performance between level 1 and levels 2 and 3 (*p*s < 0.001), making fewer mistakes on the first level than on the other two, on which their performance did not differ (*p* = 0.39).

#### Short Object Perspective-Taking (sOPT) Task and Motor Abilities

**FIGURE 3:**
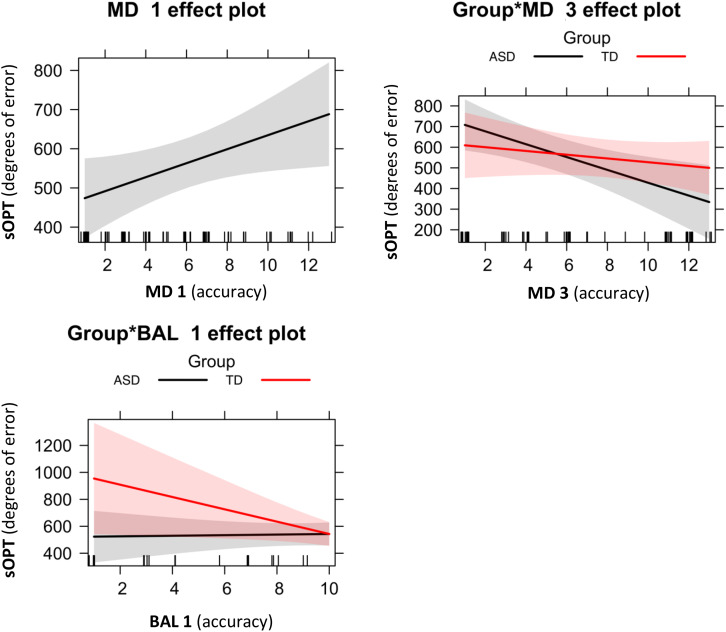
Significant effects of the best-fitting model for degrees of error in the sOPT: M3 = sOPT ∼ MD1 + Group^∗^MD3 + Group^∗^BAL1. Error bands represent 95% confidence intervals. ASD, autism spectrum disorder; TD, typically developing; sOPT, Short Object Perspective-Taking task; MD1, Manual dexterity 1; MD3, Manual dexterity 3; BAL1, Dynamic balance 1.

Following the above-described model selection strategy, as shown in Table 3, our model fitting procedure showed that the best-fitting model was M3 sOPT ∼ MD1 + Group^∗^MD3 + Group^∗^BAL1 (Figure 3). The main effects of MD 1 emerged (β = 17.87, *t* = 2.18, *p* = 0.03): shorter times to complete the MD 1 task predicted larger errors in the sOPT task. The interaction between Group and MD 3 was also significant (β = 22.03, *t* = 1.96, *p* = 0.05), showing that lower scores in the MD 3 task predicted larger errors in the sOPT task for the group with ASD, but not for the TD group. A significant effect of the interaction between Group and BAL 1 emerged as well (β = –47.89, *t* = –2.07, *p* = 0.04), showing that lower scores in the BAL 1 task predicted larger errors in the sOPT task for the TD group, but not for the ASD group.

#### Short Object Perspective Taking (sOPT) Task and Visuospatial Abilities

**FIGURE 4:**
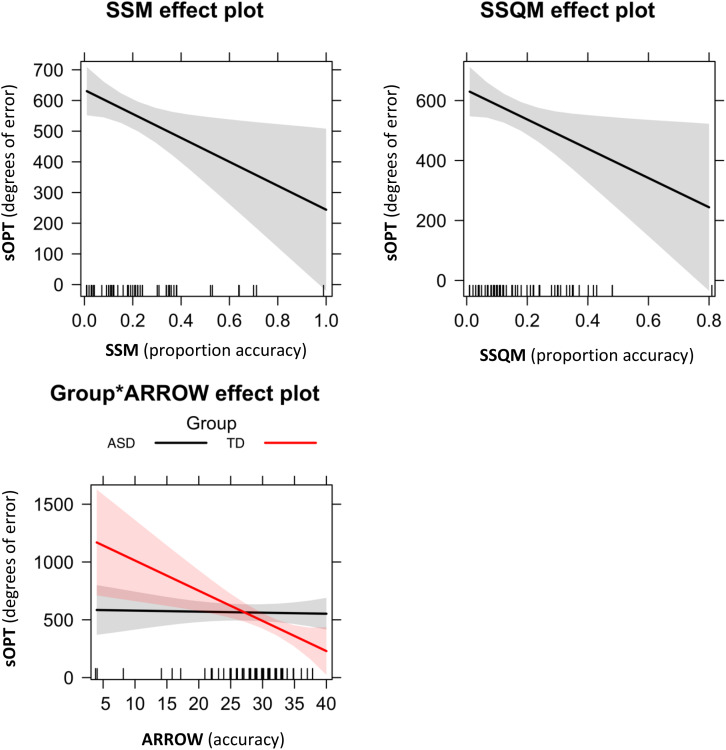
Significant effects of the best-fitting model for degrees of error in the sOPT: M6 = sOPT∼ SSM + SSQM + Group^∗^Arrow. Error bands represent 95% confidence intervals. ASD, autism spectrum disorder; TD, typically developing; sOPT, Short Object Perspective-Taking task; SSM, Spatial-Simultaneous Matrices; SSQM, Spatial-Sequential Matrices.

Concerning the association between the sOPT and visuospatial tasks, the model-fit analysis shown in Table 3 indicated that the best-fitting model was M6 sOPT ∼ SSM + SSQM + Group^∗^Arrow (Figure 4). The main effects of the SSM (β = −390.19, *t* = –2.39, *p* = 0.02) and of the SSQM (β = –488.07, *t* = –2.25, *p* = 0.03) tasks came to light. In both groups, lower scores obtained in these tasks predicted larger errors in the sOPT task. The interaction between Group and Arrow was also significant (β = –25.21, *t* = –2.64, *p* = 0.01): lower scores in the Arrow task only predicted larger errors in the sOPT task for the TD group, not for the ASD group.

## Discussion

In previous studies on typical populations, motor and visuospatial abilities revealed a crucial influence on spatial perspective-taking performance (Johnston et al., 2004; Kaiser et al., 2008; Meneghetti et al., 2012). This involvement of motor and visuospatial skills has never been studied in participants with ASD, however, and the results of studies on their spatial perspective-taking abilities have been inconsistent (Hobson, 1984; Reed and Peterson, 1990; Tan and Harris, 1991; Yirmiya et al., 1994; Warreyn et al., 2005; David et al., 2010). Since the studies were heterogeneous, the findings generated to date underscore the need to further investigate the spatial perspective-taking abilities of participants with ASD, also considering the role of any underlying processes. The present study thus aimed to examine spatial perspective-taking abilities in children and adolescents with ASD but no ID, comparing them with a group of TD peers. The influence of motor and visuospatial abilities on perspective-taking performance was also considered to shed more light on this complex visuospatial domain.

We first checked for differences in the spatial perspective-taking performance of our two groups (children with ASD vs. TD controls), taking the angular disparity of the stimuli into account. Then we looked into the role of fine and gross motor skills, and several visuospatial abilities (i.e., visuo-constructive abilities, visuospatial working memory, visual imagery, and mental rotation) in predicting spatial perspective-taking performance.

The sOPT task was used to assess our participants’ spatial perspective-taking abilities. Based on generalized mixed-effects models, both groups showed a significant effect of the angular disparity of the stimuli, showing that errors were larger greater the angular disparity. This result is consistent with previous findings (Kessler and Thomson, 2010) of individuals’ performance in spatial perspective-taking tasks worsening as the angular disparity between the egocentric and target viewpoints increased (Huttenlocher and Presson, 1973; Levine et al., 1982; Kozhevnikov and Hegarty, 2001; Zacks and Michelon, 2005, for a review). Our results also revealed differences between the two groups’ perspective-taking performance, with larger errors for the ASD group than for the TD group, but only for stimuli with an angular disparity in the range of 0–60°. There were no such differences between the groups when the task involved greater degrees of angular disparity (60–120°, 120–180°). These results partially overlap with previous reports of spatial perspective-taking abilities being intact (Hobson, 1984; Reed and Peterson, 1990; Tan and Harris, 1991; David et al., 2010) or impaired (Yirmiya et al., 1994; Warreyn et al., 2005) in participants with ASD, highlighting the influence of angular disparity. Looking at the performance of the two groups reported in Figure 2, we can see that children with ASD showed a constant worsening performance as a function of the increase of the angular disparity. Differently, the TD group performance started to deteriorate when the angular disparity increased beyond 60°, showing a preserved performance when the angular disparity was lower. Our results for the TD group are consistent with previously published findings, which indicated that the performance of TD individuals in the sOPT remained fairly constant at lower angles, then – beyond an angular disparity of around 60–90° – their performance deteriorated (e.g., Kozhevnikov and Hegarty, 2001; Keehner et al., 2006; Kessler and Thomson, 2010). No previous studies, to our knowledge, explored the effect of the angular disparity in a perspective-taking task, considering children with ASD. Although, some similarities could be drawn from the study conducted by Brunyé et al. (2012), which explored the effect of autistic traits on the perspective-taking performance of adults. They found a pattern of deterioration in performance as a function of angular deviation, particularly for adults with high ASD traits. This pattern of performance showed by Brunyé et al. (2012) was similar to the pattern showed by our children with ASD, confirming a constant deterioration of the perspective-taking performance as a function of the increase of the angular disparity.

The second aim of the present study was to see whether similar or different underlying motor or visuospatial processes might account for the two groups’ performance in the spatial perspective-taking task. To do so, we looked first at how fine and gross motor skills predicted spatial perspective-taking performance, then at the involvement of various visuospatial abilities (i.e., visuo-constructive abilities, visuospatial working memory, visual imagery, and mental rotation) in the same task.

Consistently with previous studies, our results showed that motor skills significantly affected both our groups’ spatial perspective-taking performance (Johnston et al., 2004; Kaiser et al., 2008), but not precisely in the same way. Shorter times taken to complete a manual dexterity task (MD 1) predicted larger errors in the sOPT task for both groups. Lower scores (i.e., more errors) in a manual dexterity task assessing visuomotor abilities (MD 3) also predicted larger errors in the sOPT task, but only for the group with ASD. Thus, results from the MD 3 task suggests that better fine abilities predicted better spatial perspective-taking abilities for children with ASD. Another possible explanation to consider for this result could be that both tasks require the same type of response, that is to draw. Differently, results from the MD 1 task seemed to be inconsistent with this finding. However, it is worth noting that, differently from the MD 3 task, in the MD 1 task no difference between groups emerged (see Supplementary Table S1). In this case, the role of motivational variables could be considered. Probably children did not consider the task as a challenge, perceiving it as easy and distracting. In line with what is claimed by Elosúa et al. (2017), the lack of motivation in performing the MD 1 task would have made it possible for them to get more distracted in the task. Consequently, this has led to unexpected results for this task. On the other hand, lower scores in a gross motor task assessing balance (BAL 1), based on the ability to walk forward heel-to-toe or backward toe-to-heel, predicted larger errors in the sOPT task, but only for the TD group. To our knowledge, no previous studies investigated the role of motor skills in predicting children’s spatial perspective-taking performance, but some interesting similarities with our results emerged from a study conducted by Lehmann et al. (2014) to correlate children’s motor skills, working memory and mental rotation abilities. Their results showed a positive association between balance and mental rotation abilities in TD children. Mental rotation and spatial perspective-taking abilities are known to be related (Hegarty and Waller, 2004). Judging from our results, the same is true of TD children’s balance (in terms of the ability to walk heel-to-toe or toe-to-heel) and perspective-taking abilities (Lehmann et al., 2014).

Concerning the role of visuospatial tasks in predicting spatial perspective-taking performance, a significant effect of visuospatial simultaneous and sequential working memory emerged for both our groups, showing that weaker abilities in these domains predicted greater difficulties in the spatial perspective-taking task. These results are consistent with previous reports supporting a relationship between perspective-taking ability and VSWM in the TD population (i.e., Johnston et al., 2004; Kaiser et al., 2008; Meneghetti et al., 2016; Eilam and Alon, 2019), and extend these findings to children with ASD. On the other hand, it was only in our TD children that we found a predictive effect of visuospatial processing on their perspective-taking performance, with lower scores in the Arrow task coinciding with larger errors in the sOPT task. The Arrow task assesses the ability to create and manipulate a mental representation of an object. In order to perform correctly the task, children have to imagine the path the arrow must take to get to the center of the target, considering the spatial relationships among the elements in the figure. Thus, spatial imagery abilities [i.e., the ability to represent the spatial relationships between the parts of an object and the location of objects in space or their movement (Van Garderen, 2006)] are involved in performing this task. Our result is in line with a previous report of a TD population’s perspective- taking performance being predicted by spatial visualization ability and a preference for a spatial imagery strategy (Meneghetti et al., 2012). It is worth noting that no effect of spatial imagery on perspective-taking performance emerged for our participants with ASD, suggesting that our two groups shared some visuospatial processes underlying their spatial perspective-taking performance (i.e., visuospatial working memory), but probably used different strategies. Previous research on perspective-taking suggested that different strategies might be used by children with ASD comparing them with TD children. Pearson et al. (2016) found that perspective-taking (albeit visual perspective-taking as opposed to spatial perspective-taking) was driven by differential mechanisms in these two groups. Children with TD used an embodied egocentric transformation strategy to perform a perspective-taking task. They imagined to move their own position in the space and to see the world through a different perspective. This strategy involves the ability to mentally manipulate body representations. On the contrary, children with ASD were supposed to use a mental rotation strategy, drawing on their good spatial skills. They imagined scene rotating, using a cognitive demanding spatially grounded strategy as opposed to the embodied strategy used by the children with TD. Our results are in line with the study by Pearson et al. (2016), showing that the spatial perspective-taking abilities of TD children were sustained by different processes (i.e., spatial imagery abilities) as compared with the children with ASD. We did not find the effect of mental rotation abilities on the perspective-taking performance of our group with ASD, as Pearson et al. (2016) have showed. A possible explanation for this inconsistency between the studies may relate to the different tasks used to assess mental rotation. The mental rotation task proposed by Pearson et al. (2016) used the same material of the perspective-taking task. On the contrary our mental rotation task was quite different from the sOPT. Nevertheless, both the studies suggest the importance of considering different strategies in understanding spatial perspective-taking abilities of children with ASD and children with TD, providing interesting ideas for future research.

Taken together, our findings intriguingly suggest that different abilities might be involved in explaining the spatial perspective-taking performance of children with ASD and their TD peers. Further studies will be needed to confirm and extend our results, and to overcome certain limitations of the present study, one of which concerns the small size of our samples. Given that some papers on ASD made the distinction between ASD with and without speech onset delay to account for the heterogeneity of the spectrum regarding visuospatial abilities (e.g., Nader et al., 2015; Chiodo et al., 2017), further research should take into account the effects of the speech onset delay on the perspective-taking performances of children with ASD. In addition, previous findings provided evidence for executive dysfunctions in ASD (e.g., Berenguer et al., 2018), thus it might be interesting to consider also the effect of executive functions on the perspective-taking performance of children with ASD. Finally, in order to better explain the high variability of the clinical sample, a further reflection should be made on the possibility of comparing studies that use different statistical approaches (i.e., cluster analysis or individual analysis).

We nonetheless believe that our findings shed more light on the spatial perspective-taking abilities of children with ASD as compared with their TD peers, and may help us to clarify the former’s performances in this domain. Our findings may also have some clinical and educational implications. Given the strong impact of spatial perspective-taking abilities on people’s everyday functioning – in environment learning (Allen et al., 1996; Pazzaglia and De Beni, 2006), navigation and wayfinding (Kozhevnikov et al., 2006), for instance – elucidating the strengths and weaknesses of children with ASD could lead to training activities tailored to their specific needs.

To sum up, the present findings contribute to our knowledge of the spatial perspective-taking abilities of children with ASD, how they cope with angular disparity, and in what ways they differ from their TD peers. In particular, our ASD group was relatively inaccurate at all angles, instead of reflecting the TD group’s decline in performance beyond angles of around 60°. We also confirmed the importance of examining the influence of various motor and visuospatial processes in predicting spatial perspective-taking performance as it differed in our two groups in some respects. Fine motor skills and visuospatial working memory were significant predictors for both groups, while gross motor skills and complex visuospatial abilities seemed to sustain spatial perspective-taking performance only in the TD group, not in the children with ASD. This would suggest that the two groups shared some processes but also differed in other predictors of perspective-taking performance. Hence, ASD could be considered as a form of human neurodiversity which manifests in a set of strengths and difficulties in performing a spatial perspective-taking task that may differ to the typical population.

## Data Availability Statement

The datasets generated for this study are available on request to the corresponding author.

## Ethics Statement

Ethical approval was obtained from the Research Ethics Committee at the University of Padua, Italy (protocol number: 2811). All parents had given prior written consent to their children’s participation by signing an informed consent form. Following parental consent, the participants were tested individually either at specialized centers or at their school.

## Author Contributions

RC and IM have made substantial, direct and intellectual contribution to the work. CE contributed to data analysis. All authors listed approved the work for publication.

## Conflict of Interest

The authors declare that the research was conducted in the absence of any commercial or financial relationships that could be construed as a potential conflict of interest.
