# Supplementary material for: Spatial Perspective-Taking in Children With Autism Spectrum Disorders: The Predictive Role of Visuospatial and Motor Abilities
Source: Front Hum Neurosci. 2020 Jun 3;14:208. doi: 10.3389/fnhum.2020.00208 (PMC7286568; doi:10.3389/fnhum.2020.00208)
Supplement: Supplementary file 1 [file Data_Sheet_1.PDF]

Table S1. Descriptive statistics, results of group comparisons based on one-way ANoVAs, and Cohen's d effect sizes for the measures of motor and visuospatial abilities, in children with autism spectrum disorder (ASD) and those with typical development (TD).

	ASD		TD				
	M	SD	M	SD	*F*(1, 73)	*p*	*Cohen's d*
MD 1	4.72	3.19	5.95	3.58	2.44	.122	.36
MD 3	4.77	4.11	7.77	4.03	10.11	**.002**	.73
BAL 1	8.25	3.93	10.87	1.87	13.96	**<.001**	.85
BAL 2	8.66	3.96	10.28	2.42	4.63	**.035**	.49
ROCFT	18.94	8.53	25.37	6.26	14.00	**<.001**	.86
SSM	0.19	0.22	0.22	0.16	.35	.553	.16
SSQM	0.15	0.15	0.18	0.13	.53	.468	.21
Arrows	26.47	8.24	29.21	3.78	3.49	.07	.43
AR	0.75	0.29	0.76	0.26	.15	.70	.04

Note. MD1 = Manual dexterity 1; MD3 = Manual dexterity 3; BAL1 = Dynamic balance 1; BAL2 = Dynamic balance 2; ROCFT = Rey-Osterrieth Complex Figure Test; SSM = Spatial-Simultaneous Matrices; SSQM = Spatial-Sequential Matrices; AR = Animal Rotation.

Table S2. Pearson correlation coefficients between sOPT, motor and visuospatial measures in ASD (lower diagonal) and TD (upper diagonal) groups.

Tasks	sOPT	MD 1	MD 3	BAL 1	BAL 2	ROCFT	SSM	SSQM	Arrows	AR
sOPT	1	**.358***	-.169	**-.386***	-.088	**-.372***	**-.504****	**-.502****	**-.580****	**-.398***
MD 1	.140	1	-.151	-.249	.114	**-.334***	-.288	-.099	**-.361***	-.284
MD 3	**-.458****	.154	1	-.029	-.166	.178	.161	**.579****	.060	-.063
BAL 1	-.166	.083	**.431****	1	**.423****	**.445****	**.407***	.175	**.514****	**.431****
BAL 2	-.180	-.062	-0.17	.167	1	**.595****	.252	.023	.183	.134
ROCFT	**-.522****	.203	**.382***	.170	.211	1	**.442****	.196	**.537****	**.363***
SSM	**-.603****	.080	**.340***	.053	.160	**.575****	1	**.364***	**.484****	**.324***
SSQM	**-.545****	.109	**.342***	.034	.072	**568****	**.793****	1	**.347***	.148
Arrows	-.327	**.374***	.259	.013	.154	**645****	**.471****	**.452****	1	**.354***
AR	-.274	.037	.072	.035	-.079	**.505****	**394***	**.406***	.236	1

Note. *.05 **.01
sOPT = Short Object Perspective-Taking task; MD1 = Manual dexterity 1; MD3 = Manual dexterity 3; BAL1 = Dynamic balance 1; BAL2 = Dynamic balance 2; ROCFT = Rey-Osterrieth Complex Figure Test; SSM = Spatial-Simultaneous Matrices; SSQM = Spatial-Sequential Matrices; AR = Animal Rotation.

Table S3. Skewness and Kurtosis for the residuals of each regression model.

Models	Skewness	Kurtosis
M3	-0.25	2.06
M6	0.14	2.35

Note. Skewness and Kurtosis were obtained using the “moments” package (Komsta & Novomestky, 2015), which use the following parameters for normal distribution: skewness = 0 and kurtosis = 3.
